# Dengue Virus Serotypes 1 and 2 Responsible for Major Dengue Outbreaks in Nepal: Clinical, Laboratory, and Epidemiological Features

**DOI:** 10.4269/ajtmh.17-0221

**Published:** 2017-07-31

**Authors:** Shyam Prakash Dumre, Renu Bhandari, Geeta Shakya, Sanjaya Kumar Shrestha, Mahamoud Sama Cherif, Prakash Ghimire, Chonticha Klungthong, In-Kyu Yoon, Kenji Hirayama, Kesara Na-Bangchang, Stefan Fernandez

**Affiliations:** 1 Institute of Tropical Medicine, Nagasaki University, Nagasaki, Japan;; 2 Graduate Program in Biomedical Sciences, Faculty of Allied Health Sciences, Thammasat University, Pathumthani, Thailand;; 3 Kantipur College of Medical Sciences, Kathmandu, Nepal;; 4 National Public Health Laboratory, Ministry of Health and Population, Kathmandu, Nepal;; 5 Walter Reed AFRIMS Research Unit, Kathmandu, Nepal;; 6 Tribhuvan University, Kathmandu, Nepal;; 7 Virology Department, Armed Forces Research Institute of Medical Sciences, Bangkok, Thailand;; 8 International Vaccine Institute, Seoul, Republic of Korea;; 9 Chulabhorn International College of Medicine, Thammasat University, Pathumthani, Thailand

## Abstract

Dengue virus (DENV) is expanding toward previously nonendemic areas. DENV has recently been introduced in Nepal with limited information. We report the clinical features and serotype distribution of DENV in Nepal during the 2010 outbreaks. A total of 1,215 clinical dengue cases at two major hospitals of central and western Nepal were investigated. Demographic, clinical, and laboratory parameters were recorded. Serum specimens were tested for DENV by IgM/IgG enzyme-linked immunosorbent assays (ELISAs) and reverse transcription polymerase chain reaction (RT-PCR). We confirmed DENV infection in 403 (33%) patients from 12 districts with an estimated case fatality rate of 1.5%. DENV infection was more common in adults (87%) and urban settings (74%). We detected all four serotypes but DENV-1 and -2 were mainly responsible for major outbreaks (92%). Overall, 60% of all DENV infections were secondary and 17% were severe dengue; both being more frequent among the DENV-2 infections. Rash, bleeding, abdominal pain, hepatomegaly, elevated liver enzymes, and thrombocytopenia were significantly more common in severe dengue compared with nonsevere infections. We also confirmed the expansion of dengue to hill urban areas (DENV-1 and -2), including the capital Kathmandu (altitude, 1,300 m) though > 90% cases were from southern plains. Differential clinical and laboratory features probably help in clinical decisions. Multiple serotypes circulation and elevated secondary infections pose potential risk of severe outbreaks and deaths in the future. Therefore, a country with recent dengue introduction, like Nepal, urgently requires a systematic surveillance and appropriate control measures in place to respond to any disastrous outbreaks.

## INTRODUCTION

Global distribution of dengue virus (DENV) is constantly expanding and poses a significant health problem with 390 million dengue infections/year from more than 100 countries, 96 million of which are clinical.^1–3^ These figures may still underestimate the actual dengue burden given the dramatic urbanization and inadequate dengue surveillance in tropical developing countries.^4^ The World Health Organization (WHO) South East Asia Region (SEAR) holds 50% of the global dengue burden and its member states are experiencing an upsurge in reported cases of dengue.^2,5^

DENV infections range from asymptomatic and undifferentiated fever to severe dengue manifestations. Occasionally, unusual complications such as cardiomyopathy, acute liver/renal failure, and encephalopathy/encephalitis have also been reported during dengue infections, even in the absence of severe plasma leakage or shock.^2,6^ Several studies on clinical features of DENV infections in both hospital patients and community cohorts indicate that clinical features are not uniform across the countries/continents, raising questions on their universal application in clinical settings.^7–15^ Most reports cover relatively well-resourced countries. There are few reports describing clinical features of DENV infections in resource-poor areas.

On the basis of the recent epidemiological reports, dengue has rapidly expanded to new areas (previously naïve) including Nepal.^2,3,5,16–18^ This is a global health concern and investigation in such areas provides crucial information for understanding the changing epidemiology. The first dengue outbreak in Nepal was documented in 2006, followed by a handful of sporadic reports.^16,19–21^ Due to the lack of adequate laboratory infrastructures, limited information is available on the prevailing serotypes from Nepal.^16^ Dengue remained mostly unrecognized during 2007–2009^18^ until large outbreaks occurred in central and western Nepal during 2010, at least 4 years after the first introduction of the virus in the country. The lack of data on DENV serotype, clinical manifestation, travel history of most patients and disease outcome during the 2006 outbreak has certainly left a significant knowledge gap. In this report, we describe the serological and molecular investigation coupled with demographic features of major outbreaks in Nepal to aid in understanding of regional epidemiology. Furthermore, we also sought to provide basic clinical features of DENV infections (including serotype based variation), as there is no comprehensive information available from Nepal.

## MATERIALS AND METHODS

### Study sites, patient enrollment, and specimen collection.

This observational study was conducted in two major tertiary care hospitals (Bharatpur Hospital, Chitwan in central and Lumbini Zonal Hospital, Rupandehi in western Nepal) during the large outbreak episodes to measure the dengue burden and identify the prevalent DENV serotypes along with their clinical features. Apart from southern districts, some patients from the country’s capital (Kathmandu valley) were also included in the study. A total of 1,215 febrile subjects (all age/sex) with clinical presentation similar to dengue^6^ were enrolled. A predefined set of clinical and demographic parameters were recorded on their first hospital visit and acute blood specimens were collected by veinpuncture. Selected laboratory parameters (biochemical and hematological) were recorded. Similarly, after 2 weeks, a second (convalescent) blood sample was also obtained from the subjects at hospital (in-patients) or during their follow-up visits. Days postonset of illness (DOI) was considered as the time interval between onset of fever (considered day 1) and the day of hospital visit. Outcome of the illness was also recorded.

### Definition of dengue and nondengue cases.

Dengue classification (dengue fever [DF]; dengue hemorrhagic fever [DHF]; dengue shock syndrome [DSS]) was based on the WHO guidelines of 1997.^6^ Patients’ specimens positive by dengue enzyme-linked immunosorbent assay (ELISA) and/or reverse transcription polymerase chain reaction (RT-PCR) were considered as “dengue” cases, whereas those negative by both ELISA and RT-PCR were concluded as “nondengue.” DHF and DSS were considered “severe dengue.” Information from these selected confirmed cases was used to compare clinical characteristics and laboratory parameters in dengue and nondengue populations of Nepal. For liver enzymes (serum aspartate aminotransferase [AST]; serum alanine aminotransferase [ALT]), a value > 50 IU/L was considered as “elevated.” Conditions with the platelet counts < 100,000/mm^3^ and white blood cell counts < 4,000/mm^3^ were considered as thrombocytopenia and leucopenia, respectively.

### Laboratory methods.

Each serum specimen was divided into three aliquots for serology, molecular detection and virological assays, and stored at −80°C or liquid nitrogen as appropriate.

#### DENV specific IgG and IgM ELISAs.

Specimens were assayed by DENV specific IgG and IgM ELISA (Standard Diagnostics, Inc., Yongin-si, Republic of Korea). Since the outbreak areas were previously reported as Japanese encephalitis (JE) endemic,^22^ specimens were also tested for JE using a reference standard IgM capture ELISA.^23^ There was no Zika virus (ZV) screening of these samples as no ZV has been reported in the country. In addition, malaria microscopy was also performed in these febrile cases. Immune responses (primary or secondary) were determined by previously established serological techniques (IgG/IgM ELISA) in selected patients’ specimens (both acute and convalescent sera) available in adequate quantity (*N* = 279).^23^ Additionally, as a routine, IgG and IgM rapid diagnostic test (RDT) (Standard Diagnostics, Inc.) was also used by each health facility.

#### Viral RNA detection and serotyping by RT-PCR.

**Figure 1.**
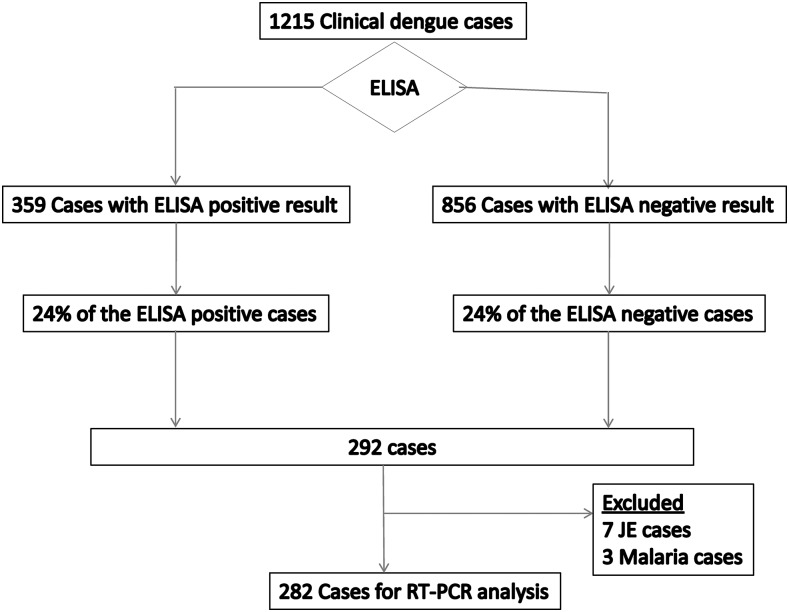
Summary of the case processing strategy and laboratory assays performed. ELISA = enzyme linked immune-sorbent assay; RT-PCR = reverse transcription polymerase chain reaction; JE = Japanese encephalitis. Among the total 3,845 febrile cases reported, 1,215 were clinical dengue cases.

Serum samples from 282 cases were randomly selected for molecular analyses, approximately maintaining the original proportion of serological dengue positive and negative population (Figure 1). DENV detection and serotyping was performed as described previously with some modifications.^24^ Briefly, viral RNA was extracted from 140 μL of patient’s serum using the commercial kit (QIAmp^®^ Viral RNA Mini Kit, QIAGEN, Germany) and amplified by first round RT-PCR using Avian Myeloblastosis virus Reverse Transcriptase (Promega, Madison, WI) in 50 μL final volume of reaction mixture containing consensus primers (D1 and D2; Supplemental Table 1). RT-PCR was done as follows: 42°C for 1 hour followed by 35 cycles of 94°C for 30 seconds, 55°C for 1 minute and 72°C for 2 minutes each. In the nested PCR step (second round), 5 μL of 1:50 diluted RT-PCR product was further amplified in a reaction mixture (50 μL final volume) containing D1 and serotype specific (TS1–4) primers (Supplemental Table 1) for 25 cycles under the same thermal conditions. Mixtures of known DENV-1 to -4 isolates and DENV-1 to -4 RNA were respectively used as positive controls for RNA extraction and PCR assays.

### Data analysis.

Mortality rate was estimated as case fatality rate (CFR) based on the deaths due to confirmed DENV. Differences in the demographic, clinical, and laboratory features between dengue and nondengue, and dengue and severe dengue populations, and outbreak sites were determined by Pearson’s χ^2^ test for categorical variables. Data distribution (normality) was determined for continuous variables by Kolmogorov–Smirnov/Shapiro–Wilk tests as appropriate. To draw the statistical difference of continuous variables in two groups and more than two groups, Mann–Whitney *U* test and Kruskal–Wallis test were used, respectively. Measures of central tendency for continuous variables were expressed as median [25–75% interquartile range (IQR)]. Statistical significance was set at *P* < 0.05 at 2-sided test statistics. Data were organized on MS Excel and analyzed by using SPSS Version 17. MapWindow GIS v4.8.6 software (Geospatial Software Laboratory, Idaho State University, Pocatello, ID) was used to locate and present the dengue cases in the country map.

### Ethical approval and informed consent.

Ethical approval was obtained from the Nepal Health Research Council (NHRC), Kathmandu, Nepal and the WHO-Ethics Review Committee, Geneva, Switzerland. Written informed consent was obtained from the subjects enrolled in the study (or their legal guardians) as appropriate.

## RESULTS

### Description of outbreak.

Beginning in mid-August 2010, a sudden upsurge in febrile cases was reported in some southern tropical districts of central and western Nepal where vector-borne diseases are endemic. Bharatpur Hospital, Bharatpur, Chitwan in central Nepal and Lumbini Zonal Hospital, Butwal, Rupandehi in western Nepal reported large number of febrile cases (*N* = 3,845) until December. Both of these public hospitals have wide catchment areas and very high patient flow as the country’s major highways pass through these two cities. These southern plains are either abundant in mosquito vectors (*Aedes aegypti* and *Aedes albopictus*) or earlier reported the presence of DENV.^16^ A notable proportion of the febrile patients (*N* = 1,215; 31.6%) were clinical dengue cases (Figure 1).

### Demographic findings.

**Figure 2.**
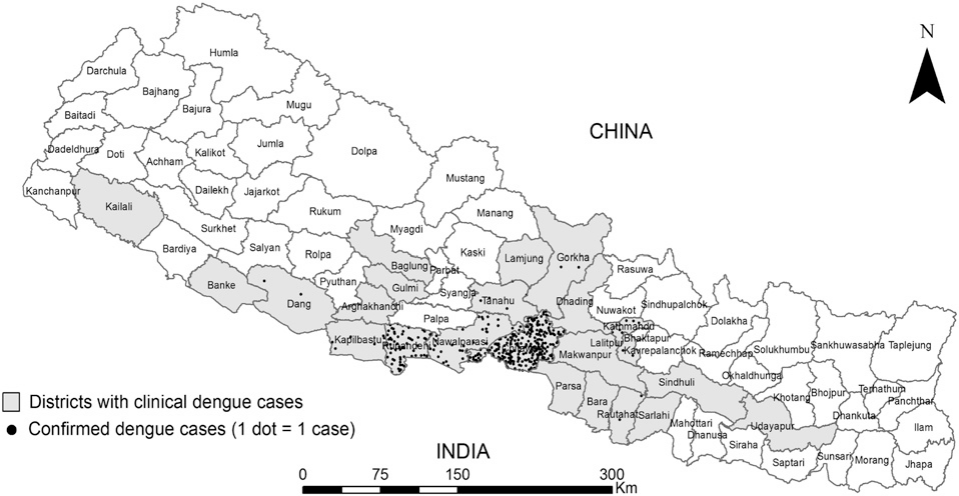
Distribution of dengue cases in Nepal. Clinical dengue cases (*N* = 1,215) were reported from 24 districts (shaded areas), and dengue virus infection was confirmed (*N* = 403) in 12 districts (dotted areas).

Out of 1,215 clinically diagnosed dengue cases reported from 24 districts during the outbreaks, 403 (33%) patients from 12 districts were confirmed to have dengue infection by ELISA and RT-PCR (Table 1, Figure 2). Seven JE and three malaria positive subjects were excluded from further analysis (Figure 1). The median age (IQR) of dengue patients was 29.5 (21.3–40.0) years, and dengue was found to be more common in males (*P* < 0.001) with the ratio (male:female) of 1.78, and among the adults (86.8%) compared with children up to 15 years (child:adult = 1:6.6) (*P* = 0.001) (Tables 1 and 2). Majority of the dengue patients (60%) had secondary infections (Table 2) and severe dengue was significantly more common in secondary infections (84%) compared with primary infections (*P* = 0.002).

**Table 1:** Demographic distribution of dengue patients, Nepal

Variable	Total cases (%)	Confirmed DENV cases (%)	Percentage of dengue positive cases (%)
Sex (*P* < 0.001)*			
Male	645 (53.1)	258 (64.1)	258/645 (40.0)
Female	570 (46.9)	145 (35.9)	145/570 (25.4)
Total	1,215 (100.0)	403 (100.0)	403/1,215 (33.2)
Age group (*P* = 0.001)*			
≤ 5	32 (2.6)	5 (1.3)	5/32 (15.6)
6–15	199 (16.4)	48 (11.9)	48/199 (24.2)
Subtotal, children ≤ 15	231 (19.0)	53 (13.2)	53/231 (22.9)
16–30	491 (40.4)	179 (44.4)	179/491 (36.5)
31–45	296 (24.4)	113 (28.0)	113/296 (38.2)
46–60	121 (10.0)	38 (9.4)	38/121 (31.4)
> 60	76 (6.2)	20 (5.0)	20/76 (26.3)
Subtotal, adults > 15	984 (81.0)	350 (86.8)	350/984 (35.6)
Total	1,215 (100.0)	403 (100.0)	403/1,215 (33.2)

DENV = dengue virus.

* *P* value was calculated using χ^2^ test.

**Table 2:** Characteristic features of dengue outbreaks in central and western Nepal

Variables	Subgroup	Central (*N* = 293)	Western (*N* = 110)	Total (*N* = 403)	*P* value
Outbreak period	–	August–December	September–December	August–December	–
Number of endemic districts	–	6	6	12	–
Serotypes	–	All 4	DENV-1, -2	All 4	–
Age in years, median (IQR)	–	30.0 (22.0–44.0)	28.0 (21–37.8)	29.5 (21.3–40.0)	0.407
Age group (years)	≤ 15	36 (12.3)	17 (15.5)	53 (13.2)	0.402
	> 15	257 (87.7)	93 (84.5)	350 (86.8)	
Sex	Male	192 (65.5)	66 (60.0)	258 (64.0)	0.303
Residence	Urban	210 (71.7)	90 (81.8)	300 (74.4)	0.041
	Rural	83 (28.3)	20 (18.2)	103 (25.6)	
Prior visit to clinics	Yes	137 (46.8)	47 (42.7)	184 (45.7)	0.469
	No	156 (53.2)	63 (57.3)	219 (54.3)	
Clinical spectrum*	DF	245 (83.6)	85 (77.3)	330 (81.9)	0.301
	DHF	45 (15.4)	24 (21.8)	69 (17.1)	
	DSS	3 (1.0)	1 (0.9)	4 (1.0)	
Immune response†	Primary	96 (48.0)	16 (20.3)	112 (40.1)	< 0.001
	Secondary	104 (52.0)	63 (79.7)	167 (59.9)	
Patient management	Outpatient	38 (12.9)	11 (10.0)	49 (12.2)	0.416
	Inpatient‡	255 (87.1)	99 (90.0)	354 (87.8)	

DOI = days postonset of illness; DF = dengue fever; DHF = dengue hemorrhagic fever; DSS = dengue shock syndrome; ICU = intensive care unit; IQR = interquartile range. Figures in the parenthesis indicate percentages unless otherwise indicated. *P* value was calculated using Mann-Whitney *U* test for age and χ^2^ test for other categorical variables.

* DHF and DSS were considered severe dengue.

† Data based on dengue patients (*N* = 279) with paired sera available for immune response determination.

‡ Inpatient data also includes 11 (2.7%) ICU patients (nine from central and two from western Nepal).

### Features of dengue outbreak in the central and western Nepal.

We further analyzed the regional variation in the outbreak pattern. Most characteristic features of the outbreak were similar in both regions (Table 2). Of the 403 dengue positive cases, the majority (293, 72.7%) were from six central districts and the rest (110, 27.3%) from six districts of western Nepal. The proportion of urban dengue was higher in the western region compared with the central (*P* = 0.041). Overall, 18.1% (73/403) of cases (48, 11.9% in central; 25, 6.2% in western) had severe dengue (DHF/DSS); however, the proportion of the severe manifestations was relatively higher (48/293, 22.7%) in western Nepal compared with central (25/110, 16.4%). Only 12% of the dengue patients were managed as out-patients while the majority (88%) required admission. The proportions of child cases, severe dengue, gender and inpatients were not significantly different in two regions. However, the prevalence of primary versus secondary infections significantly differed at the two outbreak sites (*P* < 0.001) with the proportion of secondary infections being 52.0% in central and 79.7% in western Nepal (Table 2).

### Seasonality of dengue.

**Figure 3.**
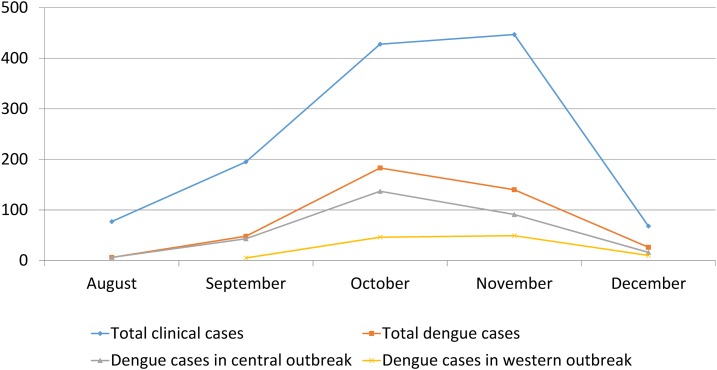
Epidemiological curve of dengue outbreaks, Nepal. Y-axis indicates the number of cases. Y-axis indicates the number of cases. Western indicates clinical dengue cases from Lumbini Zonal Hospital, Butwal, Rupandehi, and central indicates cases from Bharatpur Hospital, Bharatpur, Chitwan. This figure appears in color at www.ajtmh.org.

The dengue outbreak started on the third week of August and first week of September respectively in the central (Chitwan) and western (Butwal) Nepal (Figure 3). Ten more surrounding districts were affected within 2 months. The epidemiological curve peaked during October and November (with 34.7% and 45.4% of all cases, respectively) reflecting the dengue seasonality in Nepal (*P* < 0.001). The number of cases dwindled significantly by the end of December. Overall, the increased transmission of dengue coincided with the postmonsoon period in the country.

### Clinical and laboratory profile of dengue and nondengue patients.

Dengue patients were significantly older than nondengue patients (median [IQR] age in years = 29.5 [21.3–40.0] versus 26.0 [17.0–40.0]; *P* = 0.004) (Table 3). However, the DOI was not significantly different between dengue and nondengue febrile illnesses (*P* = 0.503). Interestingly, DOI was significantly shorter in children up to 15 years compared with adults (median [IQR] DOI in days = 3.0 [2.0–4.0] versus 5.0 [3.0–7.0]; *P* = 0.008). Detailed clinical and laboratory profiles among dengue and nondengue patient are depicted in Table 3. Most common clinical presentations in dengue patient were fever, headache, nausea, retro-orbital pain, rash, vomiting, and myalgia. Rash (*P* < 0.001), bleeding (*P* < 0.001), vomiting (*P* = 0.029), retro-orbital pain (*P* = 0.001), hepatomegaly (*P* = 0.042), elevated AST/ALT (*P* < 0.001), thrombocytopenia (*P* < 0.001) and leucopenia (*P* = 0.003) were significantly common among dengue patient compared with nondengue, whereas chills (*P* = 0.001) was more frequent among nondengue patient. Other clinical features were not significantly different in dengue and nondengue populations. Among the dengue confirmed cases, rash (*P* = 0.011), bleeding (*P* < 0.001), abdominal pain (*P* = 0.005), hepatomegaly (*P* < 0.001), elevated liver enzymes (*P* < 0.001), and thrombocytopenia (*P* < 0.001) were significantly common in severe dengue cases (DHF/DSS) compared with nonsevere dengue cases (DF). There was no difference in the frequency of severe dengue infection between children and adults (*P* = 0.187).

**Table 3:** Demographic, clinical and laboratory profile of dengue and nondengue patients, Nepal

Variable	Dengue (*N* = 128)	Nondengue (*N* = 154)	Total (*N* = 282)	*P* value
Demographic				
Age in years, median (IQR)	29.5 (21.3–40.0)	26.0 (17.0–40.0)	28.0 (19.0–40.0)	0.004
Residence, urban	93 (72.7)	65 (42.2)	158 (56.0)	< 0.001
Prior visit to clinics/pharmacy	58 (45.3)	67 (43.5)	125 (44.3)	0.761
Clinical				
DOI, median (IQR)	4.5 (3.0–7.0)	4.0 (2.0–7.0)	4.0 (2.8–7.0)	0.503
Fever	128 (100.0)	154 (100.0)	282 (100.0)	–
Headache	118 (92.2)	134 (87.0)	252 (89.4)	0.161
Nausea	91 (71.1)	97 (63.0)	188 (66.7)	0.150
Vomiting	46 (35.9)	37 (24.0)	83 (29.4)	0.029
Rash	48 (37.5)	21 (13.6)	69 (24.5)	< 0.001
Myalgia	44 (34.4)	52 (33.8)	96 (34.0)	0.914
Bleeding	18 (14.1)	4 (2.6)	22 (7.8)	< 0.001
Retro-orbital pain	56 (43.8)	39 (25.3)	95 (33.7)	0.001
Diarrhea	29 (22.7)	25 (16.2)	54 (19.1)	0.172
Sore throat	9 (7.0)	22 (14.3)	31 (11.0)	0.053
Chills	12 (9.4)	37 (24.0)	49 (17.4)	0.001
Abdominal pain	36 (28.1)	49 (31.8)	85 (30.1)	0.501
Arthralgia	19 (14.8)	17 (11.0)	36 (12.8)	0.340
Hepatomegaly	43 (33.6)	35 (22.7)	78 (27.7)	0.042
Splenomegaly	17 (13.3)	11 (7.1)	28 (9.9)	0.086
Laboratory				
Elevated AST (> 50 IU/L)	81 (63.3)	44 (28.6)	125 (44.3)	< 0.001
Elevated ALT (> 50 IU/L)	57 (44.5)	28 (18.2)	85 (30.1)	< 0.001
Thrombocytopenia	75 (58.6)	35 (22.7)	110 (39.0)	< 0.001
Leucopenia	55 (43.0)	40 (26.0)	95 (33.7)	0.003

AST = serum aspartate aminotransferase; ALT = serum alanine aminotransferase; DOI = days postonset of illness; IQR = interquartile range; IU/L = international unit/liter. Figures in the parenthesis indicate percentages unless otherwise indicated. *P* value was calculated using Mann–Whitney *U* test for age and DOI, and χ^2^ test for all categorical variables.

### DENV distribution—multiple serotypes co-circulation and expansion toward northern highlands.

We selected 282 patients’ sera (both ELISA-positive and ELISA-negative) for RT-PCR analyses (Figure 1). Overall, DENV-1 (64.4%) and DENV-2 (27.8%) were the most common serotypes found, though two cases of DENV-3 and five of DENV-4 were also confirmed (Table 4). One patient had a mixed infection with DENV-1 and -2. In the central Nepal, DENV-1 (75.0%) was found more common, whereas DENV-2 (68.2%) was the major serotype in the west (*P* = 0.001). DOI varied depending on the infecting serotypes with the shortest being DENV-1 infection (median DOI [IQR] = 3.0 [2.0–4.0]) (*P* = 0.01). Both the secondary immune response (*P* = 0.014) and severe dengue (*P* = 0.01) were significantly more frequent among DENV-2 infected subjects (Table 4). We found that dengue (DENV-1 and -2) had expanded to peri-urban and rural settings in the hill districts (Tanahun, Gorkha, Makawanpur, Kathmandu, and Lalitpur) reporting 18 confirmed cases with no travel history to endemic area for past 90 days (Figure 2).

**Table 4:** Characteristic features associated with different DENV serotypes, Nepal

		DENV-1 (*N* = 58)	DENV-2 (*N* = 25)	DENV-3 (*N* = 2)	DENV-4 (*N* = 5)	*P* value
Age in years, median (IQR)	–	29.0 (17.8–40.0)	28.0 (15.5–36.0)	ND	25.0 (20.0–37.5)	0.696
Age group (years)	≤ 15	9 (15.5)	6, 24.0	0	0	0.489
	> 15	49 (84.5)	19 (76.0)	2 (100.0)	5 (100.0)	
DOI median (IQR)*	–	3.0 (2.0–4.0)	4.0 (3.0–5.0)	ND	6.0 (4.0–6.0)	0.01
Sex	Male	41 (70.7)	15 (60.0)	2 (100.0)	3 (60.0)	0.571
Outbreak site	Western	7 (12.1)	15 (60.0)	0	0	< 0.001
	Central	51 (87.9)	10 (40.0)	2 (100.0)	5 (100.0)	
Immune response†	Primary	38 (65.5)	7 (28.0)	2 (100.0)	1 (20.0)	0.014
	Secondary	20 (34.5)	18 (72.0)	0	4 (80.0)	
Disease spectrum	DF	52 (89.7)	16 (64.0)	2 (100.0)	5 (100.0)	0.01
	DHF/DSS	6 (10.3)	9 (36.0)	0	0	

DENV = dengue virus; DF = dengue fever; DHF = dengue hemorrhagic fever; DOI = days postonset of illness; DSS = dengue shock syndrome; IQR = interquartile range; ND = not done. *P* value was calculated for age and DOI by Kruskal–Wallis test in three groups (DENV-1, -2, and -4).

* DOI difference between two groups was estimated by Mann–Whitney *U* test for DENV-1 vs. -2 (*P* = 0.023), DENV-1 vs. -4 (*P* = 0.007) and DENV-2 vs. -4 (*P* = 0.112). For categorical variables, difference was calculated using χ^2^ test. DENV-3 was not included in the statistical analysis due to small sample size (*N* = 2). Figures in the parenthesis indicate percentages unless otherwise indicated.

† Secondary infection was more common with DENV-2 compared with DENV-1 (*P* = 0.002).

### Dengue-related fatalities.

A total of 26 adult patients died during hospitalization or during the course of treatment. We detected dengue (either by RT-PCR or by combination of ELISA and RT-PCR) in six (four DHF, two DSS) of the 12 fatal cases from which we had available specimens (Table 5). This was the first outbreak with confirmed deaths due to any DENV serotypes (DENV-1 and -2) from Nepal, leaving an estimated overall CFR of 1.5% (8% in severe dengue). There were no dengue-related fatalities among children. The major complications were severe bleeding, acute organ failure, prolonged shock, and neurological manifestation. In fatal cases, ranges of time between the onset and hospital visit, and duration of hospitalization were 4–7 days and 1–7 days, respectively. Initial clinical diagnosis was poor among fatal dengue cases (50% correct diagnosis).

**Table 5:** Details of patient fatalities during the 2010 dengue outbreaks, Nepal

Patient No.	Age/Sex	DOI (day)	Hospitalized time (day)	Initial clinical diagnosis*	Dengue RDT (routine)*	Dengue ELISA	JE IgM ELISA	Dengue RT-PCR
1	56/F	7	5	Dengue	Positive	Negative	Negative	Negative
2	41/M	4	4	Malaria	Negative	Negative	Negative	DENV-1
3	27/M	8	2	Dengue	Negative	Negative	Negative	Negative
4	35/F	4	5	Leptospirosis	Negative	Positive	Negative	DENV-1
5	29/M	7	3	Dengue	Positive	Positive	Negative	DENV-1
6	14/M	2	10	UFI	Negative	Negative	Negative	Negative
7	45/M	5	6	Dengue	Positive	Negative	Negative	Negative
8	18/M	7	5	Dengue	Positive	Positive	Negative	DENV-2
9	32/F	5	2	Dengue	Positive	Negative	Negative	Negative
10	33/M	6	7	JE	Negative	Positive	Negative	DENV-2
11	21/F	4	1	Dengue	Negative	Negative	Negative	DENV-2
12	10/F	8	7	UFI	Negative	Negative	Negative	Negative

DOI = days postonset of illness; ELISA = enzyme linked immune-sorbent assay; F = female; JE = Japanese encephalitis; M = male; RDT = rapid diagnostic test; RT-PCR = reverse transcription polymerase chain reaction; UFI = undifferentiated febrile illness.

* RDT (supplied by the government) was performed by each health facility. Data on RDT and initial diagnosis was obtained from medical records.

## DISCUSSION

Here, we report the serotype distribution, clinical, laboratory, and demographic profiles of DENV infections in Nepal during 2010. This study was limited to subjects attending government hospitals. Therefore, clinical and laboratory profiles and actual picture of serotypes distribution/features are only attributable to public hospitals in Nepal.

Although we were unable to serotype all DENV positive samples, we could establish that the outbreaks were mostly caused by DENV-1 (most common in the central region) and DENV-2 (western region), a departure from the 2006 outbreak, when DENV-3 was most dominant.^16^ Also unlike previous outbreaks in Nepal, the 2010 outbreaks were characterized by the autochthonous transmission of all four DENV serotypes (no travel history among the patients) and all serotypes circulation even in a single location.

The Nepal DENV likely originated in India, given the two countries proximity and the geological, climatic, and socioeconomic similarities. This may also explain the similarities in temporal variation and seasonality between the Nepal 2010 outbreaks and contemporaneous Indian outbreaks.^25–29^ Three-fourths of the dengue cases in this study occurred in urban settings, reflecting the urban nature of the virus.^2^ Factors related to mosquito invasion, increased travel among the populace, urbanization, and globalization,^30^ and climate change^31^ have been postulated as the drivers of the expanding epidemic and could explain the introduction of DENV into Nepal and into its rural areas.^27,32^ Underscoring the expanding epidemiology of DENV is the presence of the virus in the northern hills of Nepal, including the capital city, Kathmandu (1,300 m altitude). At this altitude, DENV is infrequent as *A. aegypti* is relatively uncommon.^2^

Accurate detection of DENV required the use of both ELISA and RT-PCR (Supplemental Figure 1) rather than reliance in a single assay as results vary significantly depending on the DOI.^2^ Dengue detection rate by single serology (ELISA) and PCR was 29.8% and 31.9%, respectively. When their combination was used, the detection rate improved to 45.4%. The government of Nepal routinely provides free RDTs during outbreaks, and these are used to test single acute serum samples for diagnosis regardless of when after illness onset was the sample collected. This, combined to the RDT’s own limitations,^33,34^ leads to incorrect estimation of the burden of the disease. Unfortunately, the means to perform RT-PCR are largely lacking in Nepal and paired sera are not routinely collected in peripheral health care units. The need to improve dengue diagnosis can be addressed (to some extent) by incorporating additional early (antigen) detection assays such as nonstructural protein-1 (NS-1) detection^35^ along with serology to improve diagnosis, and facilitate proper patient management.

Early hospital visit (shorter illness duration) by urban (versus rural) residents signals their increased awareness and access to hospitals compared with their rural counterparts who substantially delay hospital attendance. Most dengue patients (88%) were hospitalized although only 18% of them were severe. Lack of laboratory facilities in resource-limited areas^15^ and inefficient classification and management experience may have contributed to unnecessarily higher hospitalization rates in Nepal. As expected, severe dengue was more common in secondary infections compared with primary.^10,11^

Characteristics of the illness varied slightly depending on the infecting serotype. Secondary infections were more commonly found in DENV-2 infections, which may have led to higher viremia in patients and more severe manifestation of the disease.^11^ Likewise, primary infections were more common among DENV-1 patients. In addition to clinical features previously described in DENV infections,^13,15^ we observed significantly higher frequency of vomiting, retro-orbital pain, hepatomegaly and elevated AST/ALT in the dengue population (versus nondengue), which are not commonly reported.^12^ Some of these clinical manifestations (rash, bleeding, abdominal pain, hepatomegaly, thrombocytopenia, and elevated AST/ALT) were more common in severe DENV infections (versus DF). Thrombocytopenia, leucopenia, and elevated liver enzymes were found significantly associated with both dengue (versus nondengue) and severe dengue (versus DF). Similar patterns are also reported elsewhere.^36–38^ These discriminatory features may be useful in patient management to allow careful monitoring for severe dengue development. Nonetheless, clinical decisions should not solely depend on these features alone. Clinical features have great value when organized into a prognostic algorithm for early prediction of dengue severity.^39^

In some countries, dengue is the leading cause of child hospitalization and death.^2,28^ Although most studies focus on pediatric dengue,^2,25,28,32^ we found more frequent DENV infections in Nepalese adults, though without differences in severity. When studies address dengue in adults,^9,10,13,26,40^ it is often in response to the virus recent introduction in an area. High frequency of adult dengue diagnosis may be attributable to lack of immunity in adults or relatively less symptomatic dengue in children.^41^ We noticed more DENV infections in males as reported before,^26,27,36^ perhaps due to higher occupational exposure to the vector among men, clothing habits and increased access to healthcare for diagnosis.^16^

Despite reports of sporadic dengue cases, dengue-associated fatalities had not been reported before 2010 in Nepal.^18^ The estimated 2010 dengue CFR was 1.5%, although only 12 of all these fatal cases were tested by RDT and of these, only six were confirmed in our study by dengue ELISA and/or RT-PCR. This CFR is slightly higher than the average fatality rate in the SEAR (∼1%).^2^ Similar to other reports.^8,9^ massive gastrointestinal bleeding and prolonged shock were the main causes of dengue-associated deaths in Nepal. Incorrect diagnosis based on clinical observations or RDTs led to treatment deviations in some fatal cases (a patient with neurological manifestations was erroneously diagnosed as JE, and nondengue case considered dengue) (Table 5). Neurological signs in dengue^42^ and treatment deviation due to misdiagnosis in fatal cases have also been reported previously.^8^ Further longitudinal and prospective studies are warranted for better understanding of dengue epidemic trends in Nepal.

## Supplementary Material

Supplemental Table.
