# Supplementary material for: Dengue Virus Serotypes 1 and 2 Responsible for Major Dengue Outbreaks in Nepal: Clinical, Laboratory, and Epidemiological Features
Source: Am J Trop Med Hyg. 2017 Jul 31;97(4):1062–9. doi: 10.4269/ajtmh.17-0221 (PMC5637613; doi:10.4269/ajtmh.17-0221)
Supplement: Supplementary file 1 [file tpmd170221.SD1.pdf]

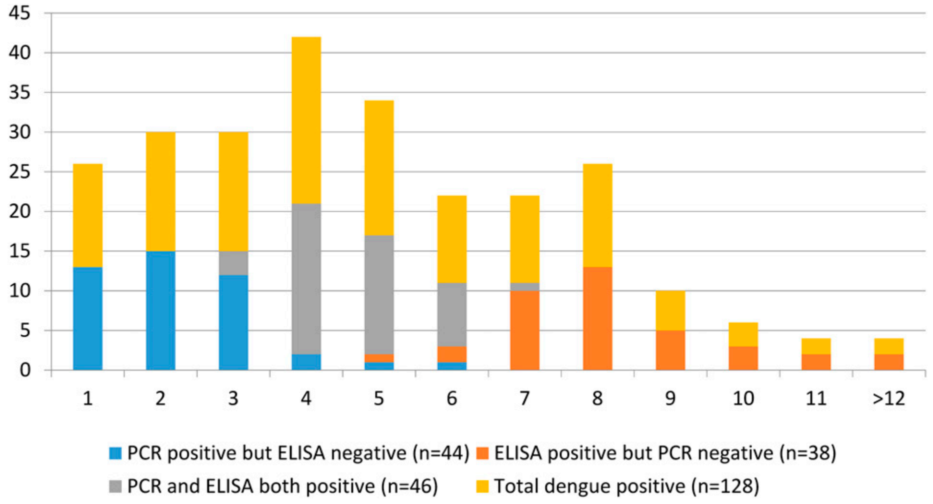

SUPPLEMENTAL FIGURE 1. Dengue detection by single serology and PCR according to postonset of illness (days). ELISA = enzyme linked immune-sorbent assay; RT-PCR = reverse transcription polymerase chain reaction. X-axis indicates days postonset of illness and Y-axis indicates number of patients.

SUPPLEMENTAL TABLE 1
Primers used in RT-PCR and nested PCR of DENV RNA for detection and serotyping

Primer name*	Amplification type	Sequence (5′→3′)	Priming position	Amplicon size in bp (primers)
D1	RT-PCR, Nested PCR	TCA ATA TGC TGA AAC GCG CGA GAA ACC G	134–161†	–
D2	RT-PCR	TTG CAC CAA CAG TCA ATG TCT TCA GGT TC	616–644	511 (D1, D2)
TS1	Nested PCR	CGT CTC AGT GAT CCG GGG G	581–599	482 (D1, TS1)
TS2	Nested PCR	CGC CAC AAG GGC CAT GAA CAG	232–252	119 (D1, TS2)
TS3	Nested PCR	TAA CAT CAT CAT GAG ACA GAG C	400–421	290 (D1, TS3)
TS4	Nested PCR	CTC TGT TGT CTT AAA CAA GAG A	506–521	392 (D1, TS4)

DENV = dengue virus; RT-PCR = reverse transcription polymerase chain reaction. These primer sequences are adapted from a previously published report.[24]
* D1 is a forward primer while the rest are reverse.
† Target priming position for D1 primer is slightly different depending upon the DENV serotype genome (118–145 for DENV-1, 134–161 for DENV-2, 132–159 for DENV-3, and 136–163 for DENV-4).
